# Quantitative stratigraphic volume of Martian lowlands using Noachian crater statistics: new bounds on volcanic effusion and flooding epochs

**DOI:** 10.3389/fspas.2025.1675588

**Published:** 2026-01-08

**Authors:** Francesco Salese, Eric Hiatt, Monica Pondrelli, Marc A. Hesse, Matilda Soldano, Alberto G. Fairén

**Affiliations:** 1 Centro de Astrobiología (CAB), CSIC- INTA, Madrid, Spain; 2 International Research School of Planetary Sciences, Dipartimento INGEO, Università D’Annunzio, Pescara, Italy; 3 Department of Earth and Planetary Sciences, University of Texas at Austin, Austin, TX, United States; 4 Oden Institute for Computational Engineering and Sciences, University of Texas at Austin, Austin, TX, United States; 5 Center for Planetary Systems Habitability, University of Texas at Austin, Austin, TX, United States; 6 Department of Astronomy, Cornell University, Ithaca, NY, United States

**Keywords:** Martian lowlands volume, lowlands stratigraphy, volatile outgassing budget from lowland igneous provinces, northern lowlands paleotopography reconstruction, Martian sediments

## Abstract

The thickness and volume of the stratigraphic sequence in Mars’ northern lowlands remain poorly constrained, despite their key role in recording the planet’s geological and paleoclimatic evolution. Reliable thickness estimates are essential because they directly control calculations of volcanic effusion, surface flooding, and associated climate forcing. Here we present a revised volumetric assessment of the lowland stratigraphy - dominated by volcanic infill - based on integrated geological mapping and crater-statistical modeling. Our approach combines crater size–frequency distributions with volumetric reconstructions of buried craters and intercrater plains across both lowland and Noachian highland reference terrains. The results indicate that the minimum cumulative stratigraphic volume is at least three times greater than previous estimates, implying a proportional increase in volcanic outgassing of CO_2_, H_2_O, and SO_2_. These new quantitative conservative bounds provide improved constraints on early Martian volatile budgets and on mid- to late-Noachian atmospheric evolution, with implications for transient climate warming and late-stage lowland flooding.

## 1 Introduction

Volcanism significantly influences the development of planetary surfaces, atmospheres, and their potential habitability. The stability of a planet’s atmosphere is intricately linked to the abundance of volatiles - including major greenhouse gases (e.g., CO_2_, H_2_O) and climate-altering species (e.g., SO_2_, H_2_S) (Jakosky and Phillips, 2001). The persistence and rate of release of these volatiles during periods of heightened volcanic activity significantly influence atmospheric composition and radiative balance, while atmospheric escape processes (e.g., photochemical loss, sputtering) act concurrently to erode the volatile inventory. Alterations in the concentration of atmospheric volatiles are considered to be among the most influential factors in determining the climatic evolution and habitability of rocky planets (Ehlmann et al., 2016).

By studying the preserved stratigraphy and geomorphology of Mars, we can gain valuable insights into early solar-system volcanism and its temporal correlation with climatic episodes. Understanding these processes on Mars also provides a comparative framework for assessing volatile budgets and atmospheric evolution on other terrestrial planets (Kopparapu et al., 2013; Kite et al., 2014). Any implication for exoplanet habitability, however, should be regarded as speculative and beyond the direct scope of this study.

The Martian lowlands are believed to have a predominantly volcanic origin in terms of their stratigraphic sequence. Nevertheless, alternative processes - including fluvial, lacustrine, and ice-related sedimentation - have contributed to the basin fill and should be considered when interpreting the stratigraphic record. While these processes locally modify the deposits, multiple lines of evidence (morphology, gravity, and surface composition) indicate that volcanic materials dominate the volume of the lowland stratigraphy (Head et al., 2002; Fuller and Head, 2002).

The gases released into the atmosphere during volcanic activity had significant impacts on the composition and thickness of the atmosphere, as well as the climate during the mid- to late Noachian and Hesperian periods. Despite these factors, the stratigraphic sequence of the lowlands remains relatively unexplored. Limited access to subsurface data has constrained previous studies, resulting in uncertainties in estimating the true thickness and volume of the lowland deposits, These limits directly affect models of volcanic outgassing, flooding, and climate evolution (Kite et al., 2017; Kite, 2018), reinforcing the role of transient warming episodes in shaping early Mars’ habitability. The present work aims to address this gap by refining volumetric estimates based on the preserved crater record, independently from previous morphotectonic approaches. In addition, we assess the main sources of uncertainty affecting these estimates, providing plausible upper and lower bounds for both the lowland infill volume and the associated volatile release. This differs from prior wrinkle-ridge and gravity-based studies by using crater population statistics as a quantitative proxy for buried topography rather than relying solely on structural or potential-field interpretations.

Determining the correlation between the volume of volcanic extrusion and the volume of volatile outgassing poses a significant challenge due to uncertainties in crucial parameters such as the total volume of extruded material and the proportion of volatiles released per unit volume of volcanic material. To tackle this issue, only one study has estimated the required volume of volcanic rocks needed to fill the lowlands. This estimation was based on the topographic expressions of wrinkle ridges, which are tectonic features formed after the cooling and solidification of lava. These wrinkle ridges were interpreted as indications of extensive Hesperian lava flows within the northern lowlands (Head et al., 2002), anchored on earlier thickness estimates by Frey et al. (2002). However, these estimates remain limited, lacking clear differentiation between volcanic and sedimentary components, as well as precise constraints on the timing and duration of volcanic eruptions (Voigt et al., 2023; Craddock and Greeley, 2009). Our study builds upon these earlier efforts but introduces a quantitative framework that integrates crater statistics with surface geology to provide conservative volumetric bounds.

The loss of atmospheric pressure and attenuation of hydrologic processes on early Mars lowered the planetary erosional rates and allowed for the retention of ancient, heavily cratered terrains created in the Noachian roughly 4 Ga ago (Golombek et al., 2006). The well-preserved Noachian terrains are found in the southern hemisphere south of the crustal dichotomy, which separates the southern highlands from the northern lowlands (Smith et al., 2001). Current stratigraphic and geophysical evidence indicates that the formation of the northern lowlands - and thus the crustal dichotomy - predates the main phase of large-basin formation. The buried lowland basement likely developed during the pre-Noachian (~4.3–4.2 Ga), while the major impact basins such as Hellas, Isidis, and Argyre formed later, between ~4.1 and 3.9 Ga (Frey, 2006; Werner, 2008). This implies that the northern lowlands were already established before most of the large impact events and subsequently underwent extensive volcanic resurfacing during the Hesperian (~3.8–3.4 Ga). In this study, we therefore assume that the lowland crust formed first, followed by the major crater-producing impacts and later volcanic infilling episodes. The relatively smooth northern plains north of the dichotomy display wrinkle ridges superimposed on a buried cratered substrate that retains crater densities comparable to exposed highlands, supporting the view of an early-formed basement later blanketed by Hesperian lavas. This implies significant volcanic infill events during early Mars history (Head et al., 2002; Fairén et al., 2003; Pan et al., 2017). The stratigraphic correlations and age constraints used in this study are informed by previous global crater chronologies (e.g., Werner et al., 2011), but the methodological details are presented separately in the Methods section.

This study focuses on deriving first-order volumetric and volatile estimates for the northern lowlands of Mars using crater statistics. We explicitly acknowledge several limitations: (1) uncertainties in the absolute age of the Noachian–Hesperian boundary; (2) limited compositional information for buried materials; (3) possible crustal deformation linked to Tharsis uplift; and (4) unquantified local effects of sedimentary or ice-related processes. While these factors introduce uncertainty, they do not undermine the overall volumetric trends discussed herein.

Estimating the infill volume required to cover the expected ancient Noachian cratered lowlands with a more recent volcanic stratigraphic sequence is crucial for assessing the associated volcanic outgassing. Accurate determination of outgassing volumes is essential to understand the evolution of the Martian atmosphere and evaluating Mars’ potential for habitability. However, previous studies have provided limited quantitative data regarding the timing, scale, and rate of volcanic outgassing events, highlighting the need for more detailed investigations. In this study, we propose a novel approach to provide a conservative baseline estimate of the past distribution of craters and the volumes of material within buried craters and their intercrater plains in the northern hemisphere of Mars. Our approach combines geological and statistical procedures to model the distribution of craters in the lowlands and the volumes of material between craters in Noachian terrains, aiming to refine volcanic volume estimates and provide clearer constraints on volcanic outgassing rates and their implications for Mars’ paleoclimate.

## 2 Geological background, aim of the study and theoretical framework

### 2.1 Geological background and focus of the study

The Martian crustal dichotomy forms the first-order boundary separating the northern lowlands from the southern highlands. Multiple hypotheses have been proposed for its origin, including a single giant impact (Marinova et al., 2008; Andrews-Hanna et al., 2008), multiple impacts (Frey, 2006), or long-term mantle convection (Zhong and Zuber, 2001).

Although its precise formation mechanism remains debated, most chronologic and stratigraphic reconstructions agree that the dichotomy and northern-lowland basement formed early - during the pre-Noachian (~4.3–4.2 Ga) - before the formation of major Noachian basins such as Hellas, Isidis, and Argyre (~4.1–3.9 Ga; Werner, 2008).

This framework implies that the lowland basin topography predates heavy bombardment and that the subsequent Hesperian resurfacing (~3.8–3.4 Ga) and later sedimentary modification only overprinted, rather than created, the original morphology (Frey, 2006; Tanaka, 2005; 2014).

The northern plains are smoother and younger than the southern highlands (Aharonson et al., 1998; Tanaka, 2005; 2014), but the mechanisms producing that smooth surface have been debated.

A range of resurfacing processes has been proposed, including fluvial/lacustrine deposition (Carr and Head, 2003; Di Achille and Hynek, 2010; Boatwright and Head, 2021), periglacial modification (Kreslavsky and Head, 2002), and ice-rich/volatiles-preserving environments (Salvatore and Christensen, 2014b).

However, several independent datasets—including gravity/topography (Zuber et al., 2000; Smith et al., 2001) and widespread mafic detections (Salvatore et al., 2010; Ody et al., 2013)- support a resurfacing dominated by volcanic infill, with sedimentary/ice-driven processes acting primarily as secondary modifiers.

Subsurface mapping of buried quasi-circular depressions (Frey et al., 2002; Frey, 2006) reveals a pre-Noachian to early-Noachian cratered basement beneath the present plains. This demonstrates that the current smooth topography does not reflect pristine basin morphology but instead burial by later infilling.

Multiple geologic observations are consistent with extensive Hesperian–Amazonian volcanic resurfacing: (1) widespread wrinkle-ridge deformation; (2) lobate flow morphologies, (3) regional topographic smoothness, (4) mafic spectral signatures.

These indicators collectively support a predominantly volcanic origin for the lowland stratigraphy (Head et al., 2002; Fuller and Head, 2002; Salvatore et al., 2010; Ody et al., 2013).

The wrinkle-ridge system is particularly significant: its amplitude and distribution are consistent with widespread emplacement of lava plains after burial of the early Noachian cratered basement - and provide a first-order lower-bound estimate of typical infill thickness.

Proposed shorelines (Clifford and Parker, 2001; Carr and Head, 2003) and multiple deltaic/fan deposits within ~200 m contouring near the dichotomy (Di Achille and Hynek, 2010) indicate that standing bodies of water may have episodically influenced lowland stratigraphy.

Similarly, large outflow channels active during the Hesperian–early Amazonian (Tanaka, 1997; Tanaka and Kolb, 2001) supplied significant sediment volumes (Carr, 1987; Chapman et al., 2003).

Aeolian and periglacial modification is also documented (Tanaka et al., 2003; Kreslavsky and Head, 2002).

However, these sedimentary contributions are volumetrically minor relative to the volcanic load: sediment estimates are at least one order of magnitude smaller than volcanic plains deposits. Their possible influence - e.g., shoreline elevation discrepancies and true polar wander (Perron et al., 2007) - introduces local variability but does not significantly affect regional volumetric mass balance.

We therefore treat non-volcanic infill as a secondary component, contributing on the order of ~20% uncertainty, which defines the upper - lower bounds used in this work.

Major volcanic centers - including Tharsis, Elysium, and Olympus Mons - have progressively resurfaced northern basins (Hartmann et al., 2000; Fuller and Head, 2002; Platz and Michael, 2011).

While the relative contributions of individual provinces (e.g., Cerberus Fossae) remain uncertain (Voigt et al., 2023), the aggregate effect is unequivocal: volcanic resurfacing is the dominant volumetric contribution to lowland fill.

This is essential because our methodology relies on reconstructing buried crater populations to estimate minimum volcanic infill thickness.

Even if sedimentary and ice-related processes locally reworked or partially insulated the substrate, their volumetric contribution is insufficient to invalidate or strongly perturb the volumetric estimates derived from crater/statistical constraints.

Thus, although multiple processes contributed to the stratigraphic evolution of the lowlands, only volcanism delivers the basin-scale mass required to bury Noachian crater populations and account for the present morphological smoothness.

In summary:

1. The dichotomy predates major impacts and establishes the initial basin architecture;
2. Volcanic resurfacing is the primary mechanism infilling that basin;
3. Sedimentary and ice-related processes are real but volumetrically limited;
4. Wrinkle ridges record the final deformation phase of thick volcanic plains;
5. Thus the lowlands’ fill volume can be quantitatively estimated from buried crater statistics;
6. Non-volcanic processes introduce limited (~±20%) uncertainty and therefore do not compromise the validity of the approach.

This provides the motivation for using crater-statistical reconstruction to quantify the total lowland fill volume and associated volatile outgassing.

### 2.2 Theoretical framework

Previous researchers have utilized qualitative methods to examine surface features buried beneath the Martian terrain (Head et al., 2002). In contrast to those approaches, which primarily relied on morphological or geophysical proxies, our method introduces a quantitative and statistically constrained framework to reconstruct the buried crater distribution and associated infill volumes. The wrinkle-ridge–based approach of Head et al. (2002) focused on identifying contractional structures and estimating resurfacing thickness from topographic relief, thus constraining post-emplacement deformation rather than the primary stratigraphic architecture. Gravity-based studies (e.g., Zuber et al., 2000; Smith et al., 2001) provided essential insights into crustal thickness and long-wavelength variations, but lacked the spatial and stratigraphic resolution required to isolate local volcanic or sedimentary fills. Similarly, sedimentary-basin–based assessments emphasized morphological basin geometry without integrating the quantitative impact crater record that constrains pre-Hesperian crustal topography (Frey et al., 2002; Frey, 2006).

Our method complements these earlier studies by integrating morphometric crater statistics with stratigraphic and compositional mapping to define conservative volumetric bounds for the lowlands. This allows us to bridge the gap between basin-scale morphological analyses and planet-wide gravity models, providing a mid-scale quantitative tool applicable to both volcanic and sedimentary infill. However, we explicitly acknowledge that our approach cannot resolve subsurface density variations or compositional heterogeneities, which remain better constrained by gravity and radar data. However, these previous studies provide a snapshot of the geological changes that have occurred on Mars. In this study, we present a quantitative approach to better reconstruct the distribution of lowlands craters in their original, unalterated morphology. Our analysis is based on the examination of early Noachian terrains that have been preserved in the highlands. To conduct our research, we made the following assumptions:

- The planetary scale dichotomy, which separates the northern lowlands from the southern highlands, is the most ancient feature on Mars. As a result, it is expected to exhibit the highest concentration of craters on the planet. Surprisingly, the plains in this region appear relatively smooth, indicating the presence of a hidden distribution of craters from the Noachian era beneath the surface (Carr and Head, 2010). Extensive evidence of Quasi-Circular Depressions (QCDs) throughout the lowlands suggests the existence of a buried population of craters (Frey et al., 2002). We therefore assume that the lowland plains from the Noachian and Hesperian ages experienced a similar rate and distribution of impacts from celestial bodies as the southern highlands, which are of comparable age. Although regional resurfacing and target heterogeneities may in principle affect crater preservation, this assumption is consistent with multiple independent datasets and published statistical comparisons, as detailed below. This assumption was tested and validated against several independent datasets and literature benchmarks. First, the crater size–frequency distributions (CSFDs) of early Noachian highlands and quasi-circular depressions (QCDs) in the lowlands were compared using global crater catalogs (Frey et al., 2002; Robbins and Hynek, 2012; Werner et al., 2011). Within the diameter range D ≥ 16 km, the cumulative frequencies and slopes of CSFDs are statistically indistinguishable at 95% confidence, confirming comparable impactor flux and crater retention for the Noachian epoch. Second, spatial variations in resurfacing were minimized by selecting highland reference areas beyond one basin diameter from Hellas and Isidis ejecta blankets (Irwin et al., 2013) and by excluding regions affected by volcanic resurfacing younger than Late Noachian (Tanaka et al., 2014). This ensures that our reference CSFDs reflect unmodified early-Noachian surfaces rather than rejuvenated terrains. Third, potential biases due to local target properties or secondary crater contamination were reduced by adopting a minimum diameter cutoff (D ≥ 5 km), above which secondary populations become negligible (Robbins and Hynek, 2012). Finally, we note that several studies (Head et al., 2002; Frey, 2006; Marchi et al., 2019) have demonstrated that buried lowland craters preserve the same cumulative crater densities as exposed Noachian highlands once resurfacing and topographic shielding are accounted for. Therefore, the assumption of equivalent impactor flux and crater density between highlands and buried lowlands is supported both statistically and physically within the temporal window relevant to this study. Potential deviations from this assumption, such as variations in impact flux or resurfacing rates between these regions, would lead to inaccuracies in the crater distribution reconstruction (De Marchi et al., 2019) and volumetric calculations.
- During the pre-Noachian period, a dichotomy formed on Mars, resulting in the lowlands having a lower elevation compared to the highlands. To reduce uncertainty related to the pre-Noachian surface, we used a minimum thickness estimate for the initial lowland elevation. We chose this conservative minimum thickness based on geological constraints provided by Head et al. (2002) and Frey et al. (2002), who estimated volcanic infill thicknesses between 1 and 2 km, acknowledging the uncertainty related to the original topographic difference prior to volcanic and sedimentary infilling.
- The fundamental assumption in crater counting and dating techniques is the presence of a stochastic impactor size-frequency distribution (e.g., Neukum et al., 2001; Platz et al., 2010b). This assumption is crucial for understanding the history of crater formation, although such estimates are subject to significant uncertainties. The cratering history in the lowlands is believed to follow this distribution, assuming it is analogous to the history observed in the highlands. In other words, the formation of large, small, and complex craters in the Noachian aged southern highlands is expected to mirror the formation of similar craters in the lowland plains. While this assumption is broadly supported by previous studies (e.g., Platz et al., 2010b; Frey, 2006), local deviations due to geological processes such as differential erosion or deposition could introduce uncertainties in crater density calculations.
- Large igneous provinces on Mars are believed to consist mainly of basaltic material, although the composition of the plains material remains uncertain. The presence of wrinkle ridges indicates that a considerable portion of the lowland fill is also likely basaltic (Lucchitta and Klockenbrink, 1981; Plescia and Golombek, 1986; Watters, 1988; 1991). Existing literature suggests that the lowlands were initially flat surfaces that were impacted and subsequently filled in with volcanic material. The estimated volume of material required for this process includes the filling of craters and intercrater plains. Observations of lobate flow fronts and proposed fissures within the plains units further support the hypothesis of a volcanic origin (e.g., Craddock and Maxwell, 1991; Head et al., 2002; Tanaka et al., 2014). A significant amount of these deposits were formed during the Hesperian period (Tanaka et al., 1988). However, alternative compositions or sedimentary contributions have been proposed in previous literature (e.g., Tanaka et al., 2003; Salvatore and Christensen, 2014b). Although we acknowledge these possibilities, we adopted a predominantly basaltic composition for consistency with previous volumetric estimates (Head et al., 2002; Craddock and Greeley, 2009).
- A potential scenario that could undermine the validity of our method involves a situation where craters do not accumulate at the same pace as they do on the reference area. This discrepancy could arise if the material composing the underlying surface of the region of interest, specifically the Martian lowlands, significantly differs in its physical properties compared to the reference surface. For instance, if the predominant composition of the region is an ice unit, it could lead to a rapid relaxation process that would diminish the number of craters and result in lower expected rim heights. However, we consider this scenario to be highly unlikely, given limited evidence supporting the occurrence of global glaciation during Mars’ Noachian period, particularly in the equatorial latitudes (see Wordsworth (2016) and references therein). Nevertheless, local ice-rich deposits or sedimentary materials with different physical properties could have contributed to localized crater relaxation, thus potentially adding uncertainty to the volumetric calculations. Another possible source of bias is viscous or viscoelastic relaxation. Although crater relaxation could, in principle, reduce rim-to-floor relief if lowlands substrates were dominated by weak, ice-rich materials, several lines of evidence indicate that regional relaxation does not bias our results. (i) Our study areas are confined to ~30° S–30° N and D ≥ 5 km, away from polar/periglacial provinces where long-wavelength relaxation is most efficient. (ii) Multiple datasets indicate that the lowlands fill is predominantly basaltic; under Hesperian conditions, kilometer-scale basaltic plains are not expected to undergo significant viscous relaxation on Gyr timescales. (iii) The widespread preservation of buried quasi-circular depressions and cratered basement signatures argues against pervasive substrate relaxation at regional scale. Operationally, our two-bracket framework already bounds any undiagnosed relaxation: the “pristine” (Garvin-based) volumes provide the upper bound, whereas the present-day MOLA-based volumes provide a lower bound that implicitly incorporates any relief reduction due to relaxation. Therefore, any relaxation would shift results toward our lower bounds without changing first-order trends or conclusions. Although localized ice-rich or weak sedimentary layers could, in principle, reduce crater rim-to-floor relief, our two-bracket framework already bounds such effects: the present-day MOLA-based volumes provide a conservative lower bound that implicitly captures any relief loss, whereas Garvin-based “pristine” volumes define the upper bound; any additional relaxation would therefore shift results only toward the lower-bound scenario, without affecting first-order trends.

To quantify the volume of lowland filling, several steps were taken. First, the potential rate of crater formation in the Noachian period (up to 3.6 Ga) and the Noachian-Hesperian period (up to 3.0 Ga) was estimated. Second, the area of the pre-Tharsis lowlands was reconstructed. Third, the rate and volume of crater formation in the lowlands were estimated. Fourthly, the volume of the filling of the intercrater plains was calculated, considering two different thickness values suggested by previous research. Finally, the contribution of Tharsis and Elysium extrusive activity was estimated.

1. The Martian geological formations that contain the oldest and most concentrated distribution of craters are found in the early Noachian aged terrains located in the Martian highlands, specifically the Early Noachian highlands units (eNh) and Early Noachian Highlands massif units (eNhmu) (Tanaka et al., 2014). Although the majority of these early Noachian terrains are situated around the Hellas impact basin, we focused exclusively on mapping the early Noachian highland units between 30°N and 28°S to minimize complications associated with ice-related resurfacing processes and latitudinal climate variations. Three specific areas within this region were selected because they represent an average among all the mapped regions. The three selected highland areas were chosen to capture the main morphotectonic and stratigraphic variability of Noachian crust while minimizing resurfacing effects. All three sectors belong to early–mid Noachian units (Npl–Nplh; Tanaka et al., 2014) characterized by stable primary crater populations and minimal volcanic resurfacing since ~3.9 Ga (Werner et al., 2011). Regions affected by ejecta blankets from large basins (Hellas, Isidis, Argyre) or by younger Hesperian volcanic flows were excluded to reduce secondary crater contamination (Irwin et al., 2013; Marchi et al., 2019). This spatial configuration - covering approximately 120° in longitude and 30° in latitude - ensures that the average crater frequency derived from these regions reflects a representative global Noachian production function rather than localized resurfacing conditions, consistent with global crater catalogs (Robbins and Hynek, 2012; Werner et al., 2011). These selected areas are among the largest single areas mapped in ArcGIS, and they strategically encompass zones located at multiples of 1, 2, and 3 inner basin ring radii, which are the expected zones of significant resurfacing around Hellas and Isidis basin ejecta (as described by Irwin et al., 2013). These areas would likely contain deposits within one basin diameter of the inner basin ring (as indicated by the light blue asterisks on the modified Supplementary Figure S4 from Irwin et al. (2013) in the Supplementary Material). Moreover, these areas are located sufficiently far from the expected zones of greatest resurfacing, thereby improving the reliability of our average measurements. By selecting these specific areas, we minimized the errors associated with reconstructing the original crater morphometry, as required by the Garvin equation (Garvin et al., 2003). The Mars geological map (Tanaka et al., 2014), was used to identify and map all craters with a diameter greater than 5 km within the aforementioned units. To estimate the combined Noachian and Hesperian crater density (number of craters per unit area), we adopted the following approach: all craters with a diameter greater than 16 km were considered to represent the Noachian crater rate, and these were combined with all craters above 5 km in diameter to estimate the integrated Noachian-Hesperian crater distribution. The choice of crater diameter was made following the crater-diameter calibration criteria of Michael (2013) and the references provided therein. This threshold selection is consistent with the reliability limits of crater chronology functions and preservation completeness in MOLA and CTX datasets (Neukum et al., 2001; Frey, 2008; Werner et al., 2011; Michael, 2013). Craters with D ≥ 16 km are statistically complete for Noachian terrains (>3.7 Ga), minimizing erasure and relaxation biases, while the D ≥ 5 km cutoff effectively captures resurfacing during the Hesperian epoch. Sub-5 km craters were excluded because production and chronology functions become strongly nonlinear at small diameters due to secondary cratering, degradation, and image-resolution limits (Robbins and Hynek, 2012). This ensures that volumetric reconstructions are based on statistically robust, morphologically well-preserved craters. The original estimate of the crater rate is nearly identical to the initial one, although an inherent margin of error exists. ArcGIS was utilized to measure the elevation of the current crater rim (h1), surrounding plateau (h2), crater floor (h), and the diameter of the crater (Figure 2). The Garvin equation (Table 1) was employed to reconstruct the original dimensions for each of these craters.
2. The estimation of the pre-Noachian lowlands surface elevation involved the elimination of the dichotomy deformation, which likely occurred due to the emplacement of the Tharsis volcanic rise (Andrews-Hanna et al., 2008). This estimation was conducted prior to the significant volcanic events that altered the morphology of the northern region of Mars. To assess uncertainty in area estimates, the estimation was performed both including and excluding the surface area occupied by the northern polar ice cap and the polar layered deposits.
3. Craters were mapped on Mars Observer Laser Altimetry (MOLA) Digital Elevation Maps (DEMs) (Smith et al., 2001) and a CTX mosaic dataset overlay (Dickson et al., 2018). Unit boundaries defined by Tanaka et al. (2014) were examined and superimposed on CTX mosaic images to estimate the areas of geologic units and crater characteristics (Dickson et al., 2018). The mapped craters were categorized into simple, complex (multi-ring crater), and basin-scale (diameter >100 km), and their original, unaltered dimensions were determined by applying the Garvin equation to calculate minimum crater volumes and surface areas per unit area. To determine crater volumes, their shape was approximated to that of a truncated cone (refer to Supplementary Figure S1 for specifics), and both the current (1) and original (2) volumes were calculated using the Garvin height. In both volume scenarios, the height considered was the difference between the elevation of the rim and the bottom of the crater, with the volume of the central peak being deemed insignificant. As illustrated in Supplementary Figure S1, volume calculation required the θ angle, determined as the difference between 90° and the slope angle. This slope was measured using the Terrain Profile Tool on 30 randomly selected craters, and the average slope angle obtained was found to be 11°, resulting in a θ angle of 79°. This subset was designed to capture the representative morphometric variability of large craters (≥5–60 km) across the lowlands rather than to provide a stratified global sample. The number of 30 craters is consistent with convergence thresholds reported in previous Mars-wide morphometric calibrations (Garvin et al., 2003; Head et al., 2002; Robbins and Hynek, 2012). Given that MOLA resolution (463 m/pixel) reliably resolves craters larger than 5 km in diameter, and that topographic slopes derived from higher-resolution CTX DEMs differ by less than 10% at these scales (Garvin et al., 2003; Robbins et al., 2014), the use of MOLA data ensures sufficient accuracy for first-order volumetric modeling.
4. The surface area of the intercrater plains was determined by calculating the combined area of the lowlands and then deducting the area covered by the craters in the Noachian and Noachian-Hesperian terrains previously calculated. To estimate the minimum volume of material necessary to fill the intercrater plains, we adopted the thickness proposed by Head et al. (2002) of approximately 1 km. This volume specifically pertains to the “Hesperian ridge unit” identified by Head et al. (2002) as filling the space between numerous wrinkle ridges in the lowlands. This thickness was selected as it represents a geologically constrained and conservative baseline based on observed wrinkle ridge topography. Frey et al. (2002) also suggested that this infill could potentially reach up to 2 km in thickness, providing an upper bound for our analysis. By exploring this range of thickness values (1–2 km), we aim to conduct a comprehensive analysis of the possible parameter space and explicitly account for uncertainties inherent to volcanic and sedimentary infilling processes.
5. The Tharsis Rise volume (enclosed by the yellow polyline in Supplementary Figure S2) was determined using the Esri ArcGIS 3D Analyst Tool Surface Volume. Areas with values ≤0 m Mars datum were excluded due to their negligible contribution to extrusive volume estimates, maintaining consistency with previous volumetric analyses by Platz et al. (2010a), Platz et al. (2011). Our calculation of Tharsis volcanic deposits, above the 0 m contour line, is 2.2 × 10^7^ km^3^, aligning closely with the overall extrusive magma volume of approximately 3.5 × 10^7^ km^3^ for Tharsis as reported by Black and Manga (2016). For comparison, the volume of volcanic material attributed to the Elysium region is sourced from literature at a value of 3.5 × 10^6^ km^3^ (Platz et al., 2010a; Platz et al., 2011).

We endeavored to calculate the minimum quantity of volatiles released during the volcanic eruptions, starting with the overall volume of volcanic material in the lowlands, and subsequently including contributions from Tharsis and Elysium. We adopted the assumption that volcanic materials are primarily basaltic in composition (bulk rock density 2700 kg-3) and that the volatile content of Martian lavas typically consists of 0.5 wt.% water, 0.7 wt.% carbon dioxide, 0.14 wt.% sulfur dioxide, and various other significant volatile constituents (Craddock and Greely, (2009) and references therein). Our goal is to provide a conservative, fundamental estimate of the lowlands volatile inventory released over time, under the assumption that basaltic volcanism was prevalent throughout Mars’ history, although the possibility of a late-stage volatile-rich pulse of andesitic volcanism cannot be ruled out. Previous studies (Carr, 1987; Head et al., 2002) suggest sedimentary deposits in the lowlands have volumes that are negligible, typically one to two orders of magnitude smaller than volcanic deposits, supporting our assumption of volcanic dominance in infill volumes.

Hereafter, a more detailed explanation:

- The Chryse basin’s large outflow channels have eroded an estimated 4.2 × 10^6 km^3^ of material to create the outflow channels and chaotic terrain, as reported by Carr (1987). This value was also referenced in the study by Head et al. (2002).
- Carr (1987) underestimated the volume of Juventae Chasma, with an estimate of 3.8 × 10^4^ km^3^. In contrast, Chapman et al. (2003) more recently calculated the Chasma volume to be 1.13 × 10^5^ km^3^, indicating a threefold difference between the two studies. Chapman et al. (2003) utilized more reliable MGS MOLA data compared to the Viking data used by Carr (1987).
- By tripling the value provided by Carr (1987), considered a minimum estimate, we arrive at a volume of 1.26 × 10^7^ km^3^, which can be considered a maximum estimate.

**Figure 1:**
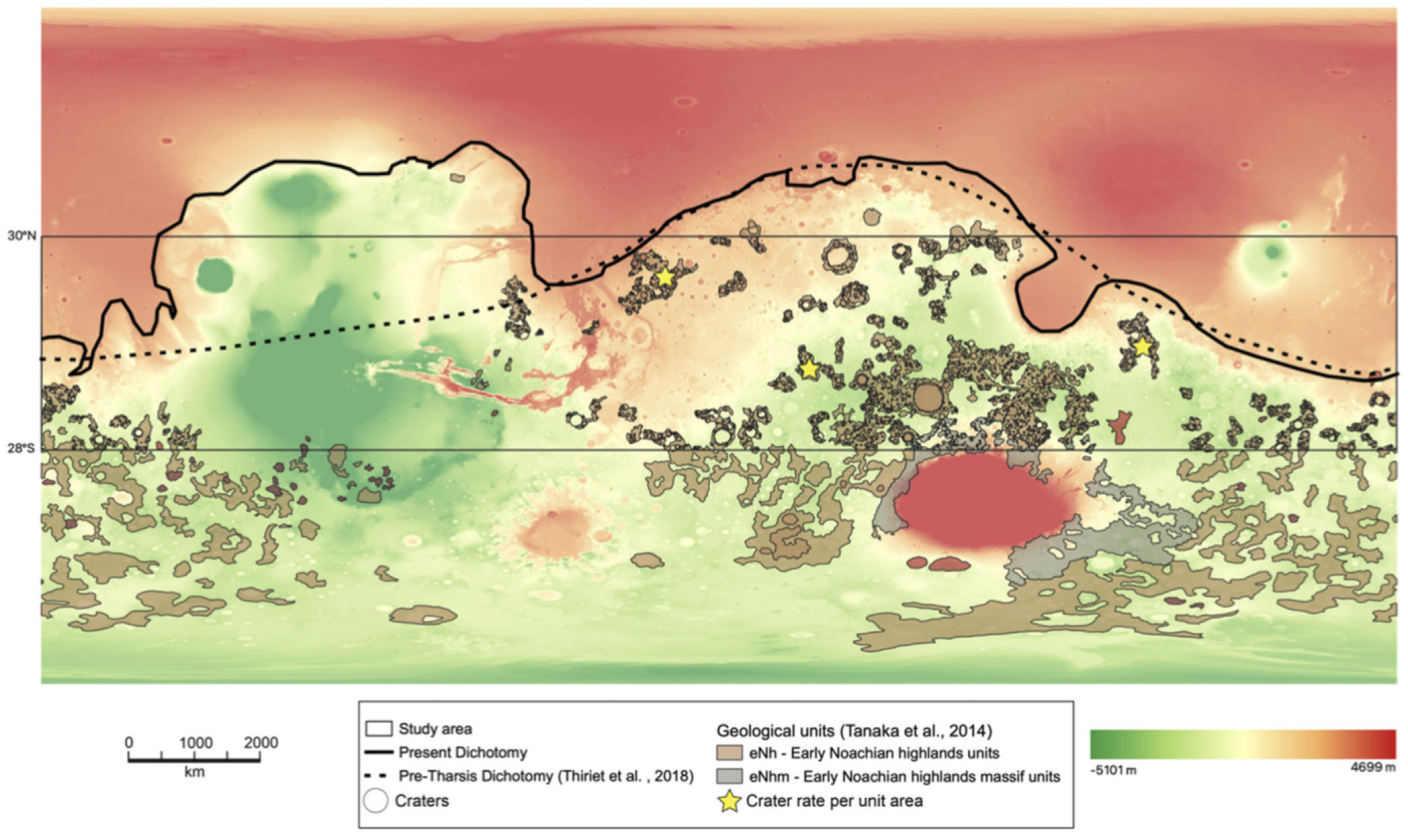
Distribution of the studied early Noachian terrains on Mars, yellow stars, selected among those mapped by Tanaka et al. (2014) and based on Mars Orbiter Laser Altimeter topography (green indicates high elevations). Their distribution follows three of the most representative highland’s areas, and they are clustered between 28°S and 30°N to avoid ice noise/erosion effects. The three selected areas are outside from the expected zones of greatest resurfacing from Hellas, Isidis, and Argyre basin ejecta, which would be within one basin diameter of the inner basin ring according to Irwin et al. (2013). Pre-Tharsis dichotomy was made following the one proposed by Thiriet et al. (2018).

The average volume of infilling in the lowlands was determined based on current MOLA and Garvin values, assuming a similar cratering rate in the early Noachian for both lowlands and highlands. This led to a comparable average volume per unit area available for infilling in the lowlands as in the highlands. The average volume available for infilling in the three selected highland areas (indicated by yellow stars in Figure 1) was calculated in relation to surface area and then extrapolated to the lowlands:

$$\begin{array}{c} \;\text{Mean}\;\text{volume}\;\text{of}\;\text{Lowlands}\;\text{infilling}\;\text{(Garvin)}\;\left[km^{3}\right]\\ =\frac{\;Volume_{1(G)}\cdot \;Volume_{2(G)}\cdot \;Volume_{3(G)}}{3} \end{array}$$

and for Mola data values

$$\begin{array}{c} \;\text{Mean}\;\text{volume}\;\text{of}\;\text{Lowlands}\;\text{infilling}\;\text{(Mola)}\;\left[km^{3}\right]\\ =\frac{\;Volume_{1(M)}\cdot \;Volume_{2(M)}\cdot \;Volume_{3(M)}}{3} \end{array}$$

Through the utilization of this method, we have estimated the total infilling value of the Lowlands through both theoretically, based on Garvin’s values, and experimentally, utilizing the obtained values along with the computation of actual shares, with Mola data. In order to proceed with this stage of the procedure, it is imperative to determine the area of the Lowlands. To achieve this, we approximate it by utilizing the Pre-Tharsis Dichotomy as the southern boundary and forming a singular polygon to encompass the Lowlands area. It is important to note that we have excluded the Tharsis and Elysium volcanic regions as well as the north Polar Cap area from our calculations. The estimated area amounts to approximately 5.32E+07 km^2^. By applying specific formulas, we tried to calculate the volume of the infilling in the Lowlands craters. The volumes were computed using both Garvin’s data and the current Mola data.

$$\begin{array}{c} \frac{\;\text{Lowlands}\;\text{crater}\;\text{volume}\;}{\;\text{Lowlands}\;\text{area}\;}=\frac{\;\text{craters}\;\text{volume}\;\text{in}\;\text{the}\;\text{cratering}\;\text{area}\;}{\;\text{cratering}\;\text{area}\;}\\ \;\text{Lowlands}\;\text{crater}\;\text{volume}\;\\ \;=\frac{\;\text{craters}\;\text{volume}\;\text{in}\;\text{the}\;\text{cratering}\;\text{area}\;\times \;\text{Lowlands}\;\text{area}\;}{\;\text{cratering}\;\text{area}\;} \end{array}$$

The following computation pertains to the intercrater region within the lowlands area. This calculation involves deducting the estimated area of Lowlands craters from the overall area of the Lowlands:

$$\;\text{Lowlands}\;\text{intercrater}\;\text{area}\;\left[km^{2}\right]=\;\text{Lowlands}\;\text{area}\;-\;\text{Lowlands}\;\text{craters}\;\text{area}\;$$

The subsequent stage involves determining the intercrater volume of the lowlands. To accomplish this, it is necessary to multiply the intercrater area of the Lowlands by its corresponding height. The height value is obtained through prior calculations and represents the greatest difference in altitude between the rim and the crater floor, thereby simulating the overall extent of the Lowlands.

Heightvalue[km]=Rimelevation-Craterfloorelevation

Even in this case the volume was calculated with Garvin data and with present Mola data:

$$\begin{array}{c} \;\text{Lowlands}\;\text{intercrater}\;\text{volume}\;[km^{3}]=\;\text{Lowlands}\;\text{intercrater}\;\text{area}\;\\ \cdot \;\text{Height}\;\text{value}\; \end{array}$$

The final stage involves the addition of the intercrater volume of the Lowlands and the volume of craters in the Lowlands, using Garvin and Mola results.

$$\begin{array}{c} \;\text{Lowlands}\;\text{infilling}\;\left[km^{3}\right]=\;\text{Lowlands}\;\text{intercrater}\;\text{plain}\;\text{volume}\;\\ +\;\text{Lowlands}\;\text{craters}\;\text{volume}\; \end{array}$$

By employing this quantitative framework, we achieve a significantly more accurate and precise estimate of the minimum volcanic infill volume in the lowlands, explicitly addressing uncertainties that previous qualitative approaches could not resolve. This enhanced precision allows us to robustly constrain conservative estimates of volatile production and associated flooding volumes, fully accounting for inherent methodological uncertainties.

Our revised minimum estimate of lowland fill volume and volcanic outgassing is anchored in the reconstructed distribution of early Noachian craters, validated via multiple independent datasets to minimize potential biases. These datasets include Context Camera (CTX) images (Malin et al., 2007), MOLA Digital Elevation Maps (DEMs) (Smith et al., 2001), and literature-derived data on contributions from Tharsis and Elysium (Platz and Michael, 2011; Black and Manga, 2016; Dohm et al., 2008).

To reconstruct the early Noachian crater distribution in the lowlands, we used elevated highland terrains (indicated by yellow stars in Figure 1; Tanaka et al., 2014) as robust proxies for the pre-infill surface, predating lowland burial before 3.6 Ga.

Their elevation above the filled plains and low erosional rates ensured excellent crater preservation, yielding a crater distribution that closely matches what would be expected north of the dichotomy (ratio A in Table 1).

**Figure 2:**
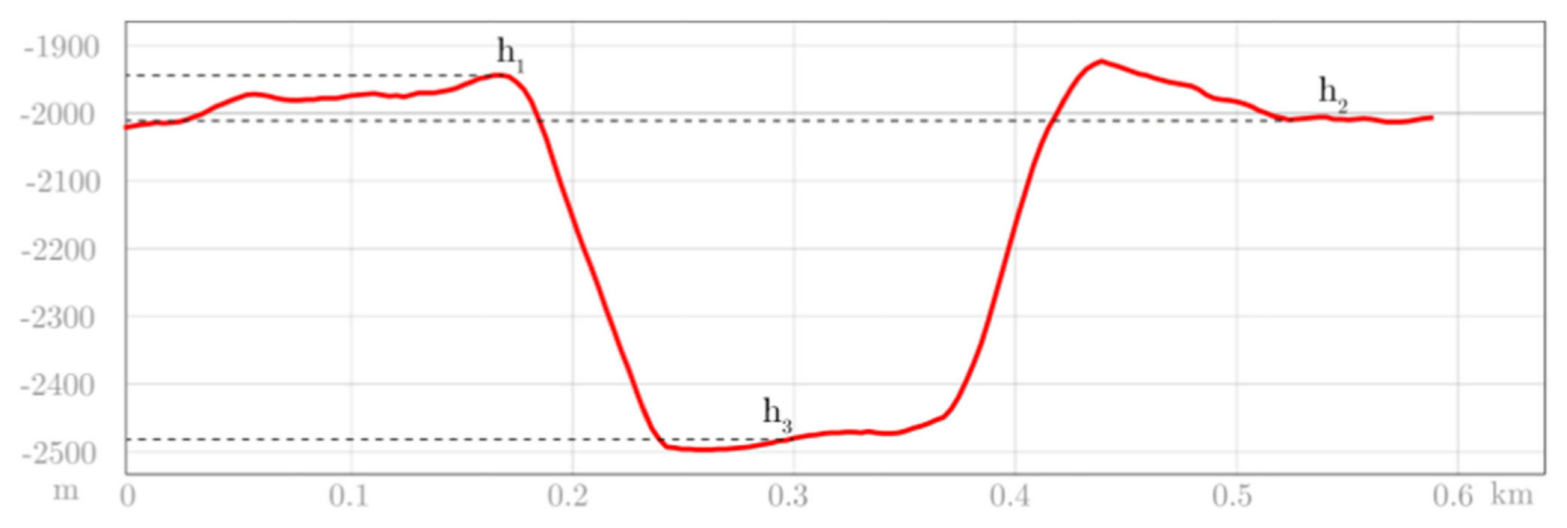
Example of topographic profile. h1 is rim elevation, h2 is plateau elevation and h3 is the crater base elevation.

Accurate quantification of available infill volume required detailed ArcGIS measurements of crater rim heights (h_1_), surrounding plateau elevations (h_2_), crater floor depths (h_3_), and diameters (Figure 2).

Using the Garvin et al. (2003) equation, we reconstructed original crater dimensions. After rigorous error analysis - including topographic variability, crater degradation, and data-resolution limitations - we proceeded to:

1. Predict crater distributions for both the Noachian (>3.6 Ga) and Noachian–Hesperian (3.6–3.0 Ga) epochs;
2. Reconstruct the pre-Tharsis lowlands paleosurface;
3. Calculate pristine crater volumes;
4. Estimate intercrater-plain infill volumes using two thickness scenarios (1 km conservative minimum from Head et al., 2002; 2 km maximum from Frey et al., 2002), conducting a comprehensive sensitivity analysis;
5. Determine extrusive contributions from Tharsis and Elysium, explicitly acknowledging uncertainties in province boundaries and literature volume estimates.

**Figure 3:**
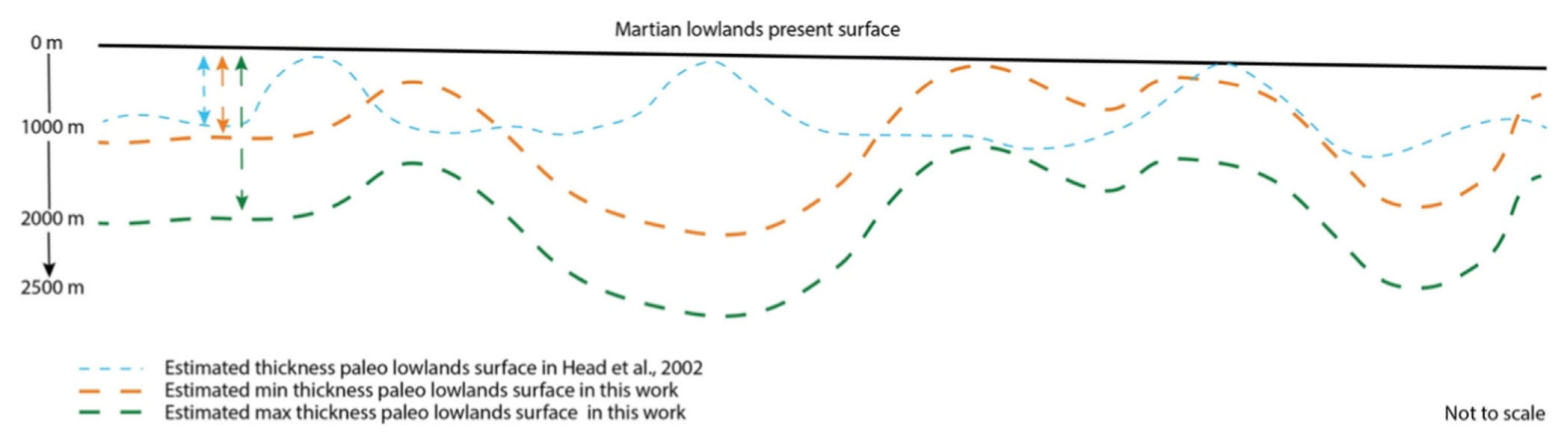
Sketch not to scale. Three hypothetical lowland surface paleoprofiles are shown here. The first profile, represented by a dashed blue line, aligns with the findings of Head et al. (2002). According to their research, the convex portions of the profile correspond to Hesperian wrinkle ridges, assuming an average thickness of approximately 1 km for the lowlands. On the other hand, the two other profiles, indicated by dashed orange and green lines, reflect the assumptions made in this study. One of these profiles considers a lowlands thickness of about 1 km, while the other takes into account an average lowlands thickness of up to 2 km. Additionally, this sketch provides a visual representation of the varying orders of magnitude in terms of volumes when compared to previous authors’ work.

In every case, crater height was defined as rim minus floor elevation; central peaks were treated as negligible. Intercrater-plain areas were computed by subtracting total cratered area from the reconstructed lowlands footprint. For the conservative infill scenario, we applied a 1 km thickness - corresponding to the Hesperian ridge unit (Head et al., 2002) - yielding the lowest bound on infill volume; the 2 km scenario provided an upper bound (Head et al., 2002) (Figure 3).

The Tharsis and Elysium rise’s extrusive volume was determined through the utilization of the Esri ArcGIS 3D Analyst Tool Surface Volume. Our calculations of the extrusive volume for Tharsis and Elysium align closely with existing literature (Platz and Michael, 2011; Black and Manga, 2016), with the exception of Elysium’s extrusive volume, which is significantly lower (order of magnitude) than that of the lowlands and Tharsis. This discrepancy does not impact the total volume significantly due to the vast difference in magnitude between Elysium and the other regions. Our assumptions regarding volcanic infill on a flat surface in the lowlands, as well as the subsequent impact and filling process, are based on prior studies (Frey et al., 2002; Head et al., 2002). We adopt a regional lowland infill thickness of 1–2 km, following the combined constraints from wrinkle-ridge relief (~0.8–1.0 km; Head et al., 2002) and the depth to buried crater rims and quasi-circular depressions (≤2 km; Frey et al., 2002). These datasets jointly define the plausible geologic range for the Hesperian volcanic plains. Thicker (>2 km) fills would exceed ridge flexural amplitudes and conflict with buried-crater geometries, hence are excluded.

To quantify the mass of key volatiles outgassed, we relied on data from literature sources (refer to (d) above) that suggest volcanic materials are primarily basaltic in composition (basalts - 2.7 g/cm^3^). The main volatile content of Martian lavas is estimated to be around 0.7 wt.% carbon dioxide, 0.5 wt.% water, and 0.14 wt.% sulfur dioxide (Craddock and Greeley, 2009), with additional minor volatile constituents excluded from this study due to their lack of relevance.

### 2.3 Uncertainty and sensitivity of the estimates

The main sources of uncertainty in our estimates arise from: (i) crater morphometry (diameter D, rim-to-floor height h), (ii) areal extent of the lowlands (A), (iii) intercrater thickness (t,1–2 km),(iv) lava density (ρ ≈ 2.7 g cm^−3^, with a plausible variability of about ±0.2 g cm^−3^ based on basaltic compositions; Head et al., 2002; Ody et al., 2013), (v) volatile mass fractions (w; CO_2_ ≈ 0.7 wt.%, H_2_O ≈ 0.5 wt.%, SO_2_ ≈ 0.14 wt.%, each varying by a few tenths of a wt.% according to Craddock and Greeley, 2009), and (vi) degassing efficiency (ε = 0.5–1.0, representing partial to complete degassing scenarios; Gaillard and Scaillet, 2009). Measurement uncertainties on D and h primarily reflect the horizontal resolution of CTX imagery and the vertical precision of MOLA topography. The range adopted for t follows the lower and upper bounds suggested by Head et al. (2002) and Frey et al. (2002), consistent with crustal-thickness estimates and buried-crater morphometry. We assessed the sensitivity of the volumetric and outgassing results by varying these parameters within their plausible geological ranges. The resulting variation defines conservative upper and lower bounds, reported as ranges in the Results section and Table 2. Parameters t and h exert the strongest control on the total infill volume, whereas ρ and w dominate the conversion of volume to gas mass. Because volumes and volatile masses scale linearly with the adopted thickness, all reported ranges can be rescaled by a factor proportional to t relative to the adopted 1–2 km interval. Extending t to 3–4 km would merely scale results proportionally and does not affect relative trends or conclusions. The adopted ranges for volatile fractions and degassing efficiency are consistent with experimental and petrologic constraints on Martian magmas (Gaillard and Scaillet, 2009; Stanley et al., 2011; Wetzel et al., 2013). Although this approach does not represent a formal statistical propagation, it captures the first-order uncertainty and provides a realistic measure of confidence in our estimates, consistent with the level of precision afforded by current Martian datasets. All ranges reported here represent plausible variability from the literature rather than analytically derived errors. Any additional reduction of crater relief by substrate relaxation would map linearly into the “present-day” lower-bound volumes and volatile masses, which we already report; thus, the published ranges encompass the plausible impact of relaxation without further modeling. Because crater and crater-population volumes scale approximately linearly with rim-to-floor relief (V ∝ h), a 10%–20% local reduction in h (as could occur over ice-rich or weak substrates) would produce a ~10–20% decrease in volume at both crater and regional scales. This effect is already encompassed by the ±20% uncertainty envelope adopted here and is by construction reflected in the difference between the present-day (lower-bound) and pristine (upper-bound) estimates. Consequently, the plausible impact of localized relaxation is quantitatively bounded within our reported ranges and does not change the inferred trends or conclusions.

## 3 The filling volume on the lowlands

**Figure 4:**
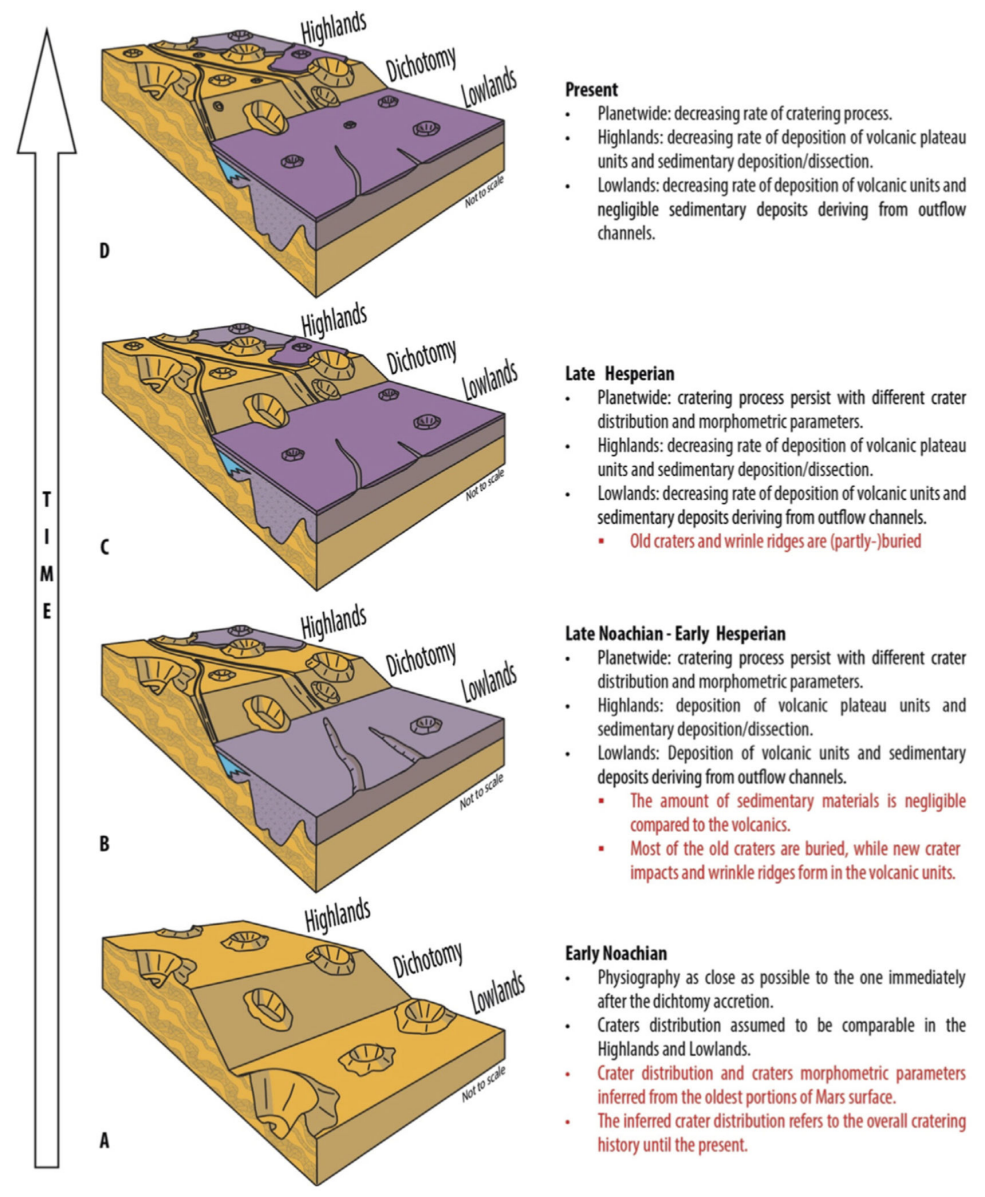
Conceptual model illustrating the evolution of Martian lowlands and their relationship with volcanic and sedimentary infilling through four chronological stages (A–D). **(A)** Early Noachian: physiography immediately following the formation of the crustal dichotomy, with comparable crater distributions in the highlands and lowlands inferred from the oldest preserved surfaces. **(B)** Late Noachian–Early Hesperian: widespread emplacement of volcanic plateau units and minor sedimentary deposits within the lowlands, leading to burial of most pre-existing craters and intercrater plains, while new impact craters and wrinkle ridges form on volcanic surfaces. **(C)** Late Hesperian: continued cratering under different distribution and morphometric conditions, accompanied by decreasing rates of volcanic and sedimentary deposition; older craters and wrinkle ridges become partly buried. **(D)** Present: modern Martian landscape characterized by a planet-wide decline in cratering rates and minimal volcanic and sedimentary infilling, with lowlands largely preserving the current surface morphology. The oldest stage is shown at the bottom and the youngest at the top; schematics are not to scale.

The surface area of the lowlands is approximately 5.2 × 10^7^ km^2^, consisting of about 30% cratered surface and around 70% intercrater plains. Our findings are influenced by factors such as filling thickness, crater density, and the state of preservation. The contribution of intercrater plains to the overall estimate is minimal. Considering the volume of intercrater plains in the lowlands, and adjusting for pristine crater volumes using the Garvin method, the minimum volume of lowlands’ fill is estimated to range between 7.9 × 10^7^ and 9.2 × 10^7^ km^3^, depending on the assumed average infill thickness and crater preservation state (refer to Section 2.3). For completeness, we note that the volumetric and volatile budgets vary linearly with the adopted fill thickness (t). The chosen 1–2 km interval is consistent with the independent constraints of Head et al. (2002) and Frey et al. (2002) and thus represents a conservative, observationally bounded range. The maximum volume of lowlands filling is estimated to be about 1.7 × 10^8^ km^3^, defining the conservative upper bound of our range. We have examined two different scenarios and eight sub-scenarios (refer to Table 2). These volumetric ranges incorporate the first-order uncertainty derived from the sensitivity analysis discussed in Section 2.3, where parameters such as infill thickness (*t*) and crater geometry (*h*) exert the strongest influence on the results. Figure 4 presents the chronological sequence of basin filling in the lowlands, starting from the Noachian period, assuming a consistent distribution rate of Noachian craters in both the highlands and the pre-Tharsis lowlands dichotomy. We consider both the morphology and volume of pristine and degraded craters. For the purpose of redefining the lowlands filling volume, our focus lies solely on volcanic craters and intercrater plains volume. We propose that the contribution of sedimentary volume (light blue in Figure 4), whether fluvial or pelagic, is relatively small compared to the magnitude of volcanic activity during the early history of Mars (Carr, 1987) (refer to Theoretical Framework). Our revised volume estimates range from two to three times the estimate (Table 2) of volcanic deposits proposed by Head et al. (2002) of 3.3 × 10^7^ km^3^.

## 4 Volatiles outgassed

The mass of each volatile species released during lowland volcanic infilling was calculated using the relation:

$$M_{v}=V\times \rho \times w_{v}\times \varepsilon$$

where *M_v* is the mass of volatile *v* (in grams), *V* is the total erupted volume of lowland infill (Section 3), *ρ* is the average lava density (2.7 × 10^3^ kg m^−3^), *w_v* is the volatile mass fraction (CO_2_ ≈ 0.7 wt.%, H_2_O ≈ 0.5 wt.%, SO_2_ ≈ 0.14 wt.%; Craddock and Greeley, 2009), and *ε* is the degassing efficiency (0.5–1.0; Gaillard and Scaillet, 2009). This formulation assumes that most volatiles were released at or near the surface and that sulfur was oxidized to SO_2_. The resulting volatile masses are summarized in Table 2. All parameters and compositional assumptions are taken from prior studies (Head et al., 2002; Craddock and Greeley, 2009; Ody et al., 2013) to ensure methodological consistency and reproducibility. Because volatile retention within melt inclusions and incomplete eruption are expected, a degassing efficiency term (*ε* = 0.5–1.0) was introduced to capture the plausible range of volatile release under Martian conditions (Gaillard and Scaillet, 2009; Wetzel et al., 2013). In addition, mid-range volatile contents adopted here (CO_2_ ≈ 0.7 wt.%, H_2_O ≈ 0.5 wt.%, SO_2_ ≈ 0.14 wt.%) are consistent with shergottite melt inclusion analyses and experimental constraints (Stanley et al., 2011; Wetzel et al., 2013).

Considering carbon dioxide as the primary atmospheric constituent, the estimated amount of carbon dioxide outgassed during the infilling of the lowlands on Mars ranges from 1.5 × 10^21^g to 3.2 × 10^21^g (Table 2). Craddock and Greeley (2009) calculated the planetary carbon dioxide budget associated with all outgassing throughout geological time to be 1.6 × 10^21^g, equivalent to around 400 mbar of atmospheric pressure. The unresolved issue pertains to the significantly larger estimate of carbon dioxide release obtained in our study, specifically associated with the infilling of the Martian lowlands alone, even though these lowlands cover only one-third of the planet’s surface. Our calculated CO_2_ output thus exceeds previous estimates for the entire planetary volcanic activity by at least two to three times. This discrepancy may arise from uncertainties in magma volatile content, the completeness of degassing, and the methodological differences in estimating volcanic volumes and eruption frequencies, similar to uncertainties described in volcanic degassing studies on Mercury (Kerber et al., 2009) and the Moon (Needham and Kring, 2017).

A comparison can be made between the current amount of carbon dioxide in the atmosphere, which is approximately 2.5 × 10^19^ g (Zuber et al., 2007), and the estimated release of juvenile water from volcanism onto the atmosphere/surface of Mars, ranging between 1.1 × 10^21^g and 2.3 × 10^21^g. Previous research suggested that about 1.1 × 10^21^g (8 ± 1 mm^-2^) of juvenile water has been released over Mars’ entire geologic history, with more than half of this quantity now found in the north polar cap and the atmosphere (Craddock and Greeley, 2009) (Table 2). Our calculations yield an equivalent quantity based primarily on the volcanic infilling of the lowlands. However, given that this region represents only a fraction of Mars’ volcanic history, previous estimates must underestimate the planet’s total juvenile water release because our work illustrates that the filling of the northern lowlands supplied these volumes despite being a fraction of Mars’ volcanic history. Future studies should account for this broader geological context to refine total planetary volatile budgets.

Additionally, we have approximated the release of SO_2_ to be within the range of 3.0× 10^20^ to 6.4 × 10^20^g (Table 2). The presence of SO_2_ impacts the overall atmospheric pressure on a sub-decadal timescale and transforms into SO_3_ in the presence of hydrogen species, a transformation that is directly linked to the atmospheric water content (Craddock and Greely, 2009). Following precipitation, this volatile substance is reintroduced to the planet in the form of acid rain (H_2_SO_4_). Furthermore, SO_2_ serves as a potent greenhouse gas and could have been a significant component of the Martian atmosphere during periods of heightened volcanic activity (Postawko and Kuhn, 1986). Given the demonstrated significance of volcanic SO_2_ on other planetary bodies, such as Mercury (Kerber et al., 2009), the Moon and icy satellites exhibiting cryovolcanism (Lopes et al., 2007; Menten et al., 2022; 2024), we emphasize the importance of further constraining SO_2_ degassing rates and their climatic impact through detailed atmospheric modeling. These SO_2_ volatiles may also contribute to the warming of Mars as a secondary factor and play a crucial role in various atmospheric models (Kasting, 1991; Postawko and Kuhn, 1986).

## 5 Lowlands volcanic flooding timing

The widespread volcanic flooding observed on Mars suggests the intrusion of magma into the crust during the Noachian-Hesperian period, which dates back to around 3.0 billion years ago. If Mars had a solid lid, it is possible that partially melted mafic and ultramafic rocks within the upper mantle could have formed and moved through dike propagation to magma chambers or directly to the surface (O’Neill et al., 2007; Ehlmann and Edwards, 2014). Geodynamic modeling of the Martian mantle has indicated a change in the rate of magma emplacement over time, ranging from 0.11–0.24 km^3^, with a best estimate value of 0.17 km^3^ yr^−1^, per year during the early Hesperian period to approximately 1 × 10^−4^ km^3^ yr^−1^ at present (O’Neill et al., 2007). Based on our analysis, which estimates a maximum volume of magma emplacement in the lowlands at 1.7 ± 0.05 × 10^8^ km^3^ and a magma emplacement rate of 0.17 km^3^ a^−1^per year, we calculate that the volcanic flooding in the lowlands would have taken approximately 0.6 billion years to occur, spanning a significant portion of the late Noachian to Hesperian periods. The presence of the Tharsis volcanic province, with differing theories on its emplacement timing, suggests that the Noachian-aged craters in the lowlands were filled and buried by subsequent volcanic activity (Greeley and Schneid, 1991; Phillips et al., 2001). While our estimates do not provide insights into the transient mechanisms of a “wet and warm” climate excursion, they are valuable for studying the loss mechanisms of planetary volatile budgets. This estimation serves as a constraint on the cumulative loss rate.

## 6 Implications

This study emphasizes the significance of employing a geological approach and conducting thorough analysis when estimating the emissions of lava and volatiles. While we acknowledge the challenges associated with estimating the cumulative extrusion of volatiles, it remains essential for climate models and overall assessments of atmospheric volatile budgets. Our analyses indicate that comprehending the quantity of lava that filled the lowlands can greatly enhance paleoclimate models and processes spanning from the early Noachian to Hesperian periods. Moreover, our theoretical framework presents a promising technique for redefining the amount of Martian lava erupted through the utilization of remotely sensed images. However, despite our best efforts to reconstruct the distribution of lowlands craters and intercrater volume accurately, determining the exact volume of the lowlands necessitates further experimental and theoretical evaluation that cannot be conducted with the current instruments available on Mars. To validate our model, gravity survey missions or underground investigations are imperative. Despite these limitations, our approach is complementary to previous morphotectonic and gravity-based studies: while gravity surveys capture deep crustal architecture, our crater-statistical framework resolves the volumetric consequences of surface burial processes. Together, these methods provide a coherent multi-scale picture of the formation and modification of the Martian lowlands.

The volumetric and outgassing estimates presented here represent time-integrated cumulative volatile budgets associated with the volcanic infilling of the northern lowlands. Because the derived volumes are based on crater-retention and morphological constraints rather than eruption chronologies, they do not resolve the exact temporal spacing of individual eruptions. However, geomorphological and stratigraphic observations suggest that lowland volcanism was episodic and pulsed rather than continuous. The occurrence of wrinkle-ridge deformation, interbedded lava sequences, and regionally variable resurfacing ages (Head et al., 2002; Platz and Michael, 2011; Brož et al., 2021) indicates multiple eruptive episodes spanning the Late Noachian to Late Hesperian, separated by quiescent intervals. Such pulsed degassing would have generated transient increases in atmospheric pressure and temperature, followed by cooling and volatile loss phases. After each major eruptive pulse, atmospheric escape processes - including photochemical dissociation, solar-wind sputtering, and ion pick-up - would have progressively removed CO_2_, H_2_O, and SO_2_ from the atmosphere (Lammer et al., 2013). These escape mechanisms, combined with surface adsorption and limited crustal carbonate formation, imply that the calculated volatile masses should be regarded as upper limits to the total degassed inventory rather than the retained atmospheric content. Consequently, our results constrain the cumulative magnitude of volatile release from lowland volcanism, whereas the temporal dynamics of eruption and the efficiency of atmospheric escape determined the fraction of volatiles effectively retained through time.

## 7 Conclusion

Through the reconstruction of the early Noachian highlands pristine craters distribution, our theoretical framework presents a robust proxy for analogous lowland crater distributions. We present a novel lowland filling volume estimate - approximately three times higher than prior assessments - which in turn yields more precise quantifications of primary volcanic volatiles (CO_2_, H_2_O, SO_2_). These findings bear profound consequences for understanding Mars’s volatile evolution, climate history, and potential habitability.

## Figures and Tables

**Table 1:** Ratio between intercrater plains and crater surfaces of the three studied areas. The values of each area and the final average show that the selected areas are consistent and representative.

Highlands test zone	Ratio A (intercrater plains surface/crater surface)
1	1,822 ± 0.002
2	2,967 ± 0.002
3	2,301 ± 0.003
Average	2,363 ± 0.007

**Table 2:** Summary of the two proposed scenarios and the related 8 sub-scenarios: 4 sub-scenarios for scenario 1 and another 4 for scenario 2. Scenario 1, which is the one with the highest values both for the filling volume of the lowlands and for the gases emitted, was determined using the values reconstructed with the Garvin equation for the dimensions of the craters, while scenario two assumes the values of the dimensions of the current craters thus also considering the phenomena of weathering.

					H2O (g)	CO2 (g)	SO2 (g)	Assumed density (g/cm^3^)
Scenario this work	Sub-scenario	Lava volume (km^3^)	Lava volume (cm^3^)	Lava Mass (g)	0.005	0.007	0.0014	2,7
1	1 - MAX	1.70E+8	1.70E+23	4.6E+23	2.3E+21	3.2E+21	6.4E+20	
1	2	1.27E+8	1.27E+23	3.4E+23	1.7E+21	2.4E+21	4.8E+20	
1	3	1.24E+8	1.24E+23	3.3E+23	1.7E+21	2.3E+21	4.7E+20	
1	4 - MIN	0.92E+8	0.92E+23	2.5E+23	1.2E+21	1.7E+21	3.5E+20	
2	1 - MAX	1.54E+8	1.54E+23	4.2E+23	2.1E+21	2.9E+21	5.8E+20	
2	2	1.15E+8	1.15E+23	3.1E+23	1.6E+21	2.2E+21	4.3E+20	
2	3	1.11E+8	1.11E+23	3.0E+23	1.5E+21	2.1E+21	4.2E+20	
2	4 - MIN	0.79E+8	0.79E+23	2.1E+23	1.1E+21	1.5E+21	3.0E+20	
Craddock and Greely (2009) Whole mars		-	-	-	1.1E+21	1.6E+21	-	2,7

The two scenarios also differ in the thickness taken into consideration for the filling of the lowlands, respectively 2 and 1 km. The details of each single scenario are explained in the tables attached in the Supplementary Methods. For each scenario, a minimum value, a maximum value and two intermediate values of the volume of volcanic material that filled the lowlands are indicated, as well as the volume of the main gases released. At the bottom of the table is shown the value of the two main gases estimated by Craddock and Greely (2009) for comparison, the values previously estimated by the authors are for the whole Mars while ours are only for the Lowlands, i.e., just over one-third of the planet’s surface.

## Data Availability

The original contributions presented in the study are included in the article/Supplementary Material, further inquiries can be directed to the corresponding author.

## Highlights

- First quantitative assessment of the thickness and volume of the Martian northern lowland stratigraphic sequence, integrating crater-distribution statistics with intercrater-plain morphology.
- Revised lowland thickness increases total buried volume by at least a factor of three, reshaping estimates of volcanic effusion, paleoflooding, and climate evolution.
- Derived CO_2_, H_2_O, and SO_2_ release from these updated volumes constrains the time-integrated volatile budget and early atmospheric evolution of Mars.

## 8 Plain language summary

Our knowledge of the thickness and volume of the geological layers beneath Mars’s northern lowlands remains limited, even though these buried deposits record key stages of the planet’s geological and climatic history. Accurately estimating their thickness is important: if the layers are thicker than previously thought, it means that much more volcanic material once filled the lowlands, releasing larger amounts of gases and possibly allowing surface water to accumulate long ago. In this study, we introduce a new method that combines surface geology with crater statistics to estimate how much material lies beneath the northern plains. By comparing how many impact craters should have been buried by lava flows with their preserved shapes, we calculate the total volume of volcanic infill. Our results show that the buried volume is at least three times greater than earlier estimates. This suggests that early Mars may have released far more CO_2_, H_2_O, and SO_2_ into its atmosphere, providing new clues about how the planet’s climate briefly warmed and how its atmosphere later became thin and dry.

## Footnotes

**Supplementary material**
